# From the Editor's desk

**DOI:** 10.4103/0970-2113.56339

**Published:** 2009

**Authors:** S. K. Jindal

**Affiliations:** *Lung India, E-mail: skjindal@indiachest.org*

I am glad to say that the first year of our online submission, review and publication process completes with this issue. This issue contains all the pending articles that had been accepted for publication in the past. The remaining articles in the various stages of the reviewing process, which were received as hard copies, have been returned to the authors with the request for online submission.

The online submission process has been fairly satisfactory for all of us. This has not only quickened the process but also helped in the improvement of the quality of the journal. We hope to improve it further with the addition of more articles and other features.

I seek a greater involvement and interest of the members of the Indian Chest Society to make this journal as influential and important in the overall health scene in this country. All members, particularly those with academic pursuits need to provide more inputs for the success of Lung India. Sharing knowledge and ideas with others is always satisfying. I may quote from the age-old wisdom – “As one lamp lights the other – nor glows less!” I may therefore request everyone to devote a little time and knowledge for the benefit of the journal. This will greatly help in the general growth of pulmonary sciences.

I also invite your greater participation in the reviewing process of the journal. Those interested in this process are requested to write to us for inclusion of their names.

I am sure that with you all along, we shall continue to march forward.

